# An Investigation of Sintering Parameters on Titanium Powder for Electron Beam Melting Processing Optimization

**DOI:** 10.3390/ma9120974

**Published:** 2016-12-01

**Authors:** Philipp Drescher, Mohamed Sarhan, Hermann Seitz

**Affiliations:** Fluid Technology and Microfluidics, University of Rostock, Justus-von-Liebig Weg 6, 18059 Rostock, Germany; mohamed.sarhan@uni-rostock.de (M.S.); hermann.seitz@uni-rostock.de (H.S.)

**Keywords:** additive manufacturing, electron beam melting, sintering, efficiency, powder removal

## Abstract

Selective electron beam melting (SEBM) is a relatively new additive manufacturing technology for metallic materials. Specific to this technology is the sintering of the metal powder prior to the melting process. The sintering process has disadvantages for post-processing. The post-processing of parts produced by SEBM typically involves the removal of semi-sintered powder through the use of a powder blasting system. Furthermore, the sintering of large areas before melting decreases productivity. Current investigations are aimed at improving the sintering process in order to achieve better productivity, geometric accuracy, and resolution. In this study, the focus lies on the modification of the sintering process. In order to investigate and improve the sintering process, highly porous titanium test specimens with various scan speeds were built. The aim of this study was to decrease build time with comparable mechanical properties of the components and to remove the residual powder more easily after a build. By only sintering the area in which the melt pool for the components is created, an average productivity improvement of approx. 20% was achieved. Tensile tests were carried out, and the measured mechanical properties show comparatively or slightly improved values compared with the reference.

## 1. Introduction

Selective electron beam melting (SEBM) shows a high capability for the fabrication of complex net-shaped titanium parts for technical and medical applications. In the medical field, it is highly suitable for the manufacturing of implants since SEBM is able to fabricate designed and controlled porosity and tailored surface quality for the purpose of better bone growth. In regard to mechanical properties, studies have shown that titanium parts made via SEBM are on the same level as its conventional alternatives [1,2,3,4,5].

The SEBM technology, as many other additive manufacturing technologies, uses powder as the raw material to build three-dimensional structures [6]. The use of powder has some advantages but also disadvantages, such as its removal from the parts. Contrary to laser melting systems, SEBM-manufactured parts are embedded in a semi-sintered powder. The powder recovery system (PRS) from the company Arcam AB, Sweden, is a sandblast technology that uses powder from the build job for powder removal, simultaneously recycling and refurbishing it. Since powder removal is a vital part of the post process, it has already been investigated in previous studies [7,8].

In order to use a high-energy electron beam for melting, the metal powder needs to be semi-sintered in a previous process in order to avoid repulsion effects caused by the electrons [9]. The semi-sintered powder bed is achieved by using two large area preheat strategies. The first preheat strategy is necessary for anchoring the particles strongly enough in order to move a higher energy electron beam without the undesired repulsion effect. This is called “jump safe”. The second preheat strategy uses a high energy electron beam and is necessary for anchoring the particles even further in order to make the powder bed “melt safe”. The melting process uses even higher energy densities so that the material can reach melting temperature.

There have been some investigations on changing the parameters in order to achieve a better understanding of the sintering and melting process [10,11,12]. This can lead to improved electron beam melting processes and resulting mechanical properties of manufactured components. A modified preheat strategy can be used, for example, to build components with similar mechanical properties but with faster build time.

The aim of this paper was to apply different preheat strategies in order to investigate the sintering quality of the powder and the feasibility for a modified melting strategy. The procedure was to replace the second large area preheat strategy with a strategy that only sinters the area to be consecutively melted. By doing this, the surrounding powder is not sintered as strongly and the finished parts can therefore be removed more easily and the build process time can be decreased, resulting in a higher productivity.

## 2. Materials and Methods

**Figure 1:**
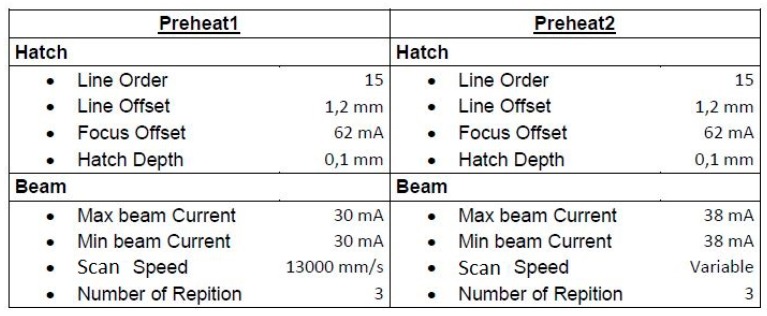
Initial preheat parameters with the variable scan speed for Preheat2.

The SEBM system A1 of the company Arcam AB, Mölndal, Sweden, was used with the metal powder Ti6Al4V. The spherical powder has a particle size distribution of 45–105 µm and was acquired from the company Arcam AB. The samples were built with a layer thickness of 50 µm. The sintering parameters were customized in order to achieve an increased sinter quality. A test series was carried out in which different scan speeds for Preheat2 were used for sintering only the area of the components. The parameters for Preheat1 were left unchanged since this is a critical process for the sintering of the loose powder to create an initial stability. In this regard, Preheat2 continues on pre-sintered powder and has therefore a larger process window. For this study, Preheat2 was modified by the variation of the scan speed. The test series was carried out in order to investigate the increase in the sintering quality of the powder. The starting scan speed was the established scan speed of 14.6 m/s from the standard Preheat2 strategy of the preheat theme. Going from there, a decrease in scan speed by 1 m/s was implemented until a scan speed of 8.6 m/s was reached. The following general preheat parameters (see Figure 1) were used for the test series.

After the preheat theme, the melt theme was used with the standard parameters of the 50 µm layer thickness theme. The power and diameter of the electron beam changes during the processes due to different algorithms, but has a range of 0–50 mA and is roughly 100–500 µm, respectively.

For the investigation of the influence of parameter variation on mechanical properties, test samples were built. The test samples consist of sinter cake cylinders with different scan speeds for compressive testing as well as fully melted flat tension bars for tensile testing.

The cylinders had a diameter of 20 mm and a height of 40 mm. The compression test was carried out in regard to the standard DIN 50106 with a test speed of 60 mm/min. At least 10 samples were tested for statistic accuracy. The tests were carried out with the universal test machine Zwick/Roell Z5.0 of the company Zwick GmbH & Co. KG, Ulm, Germany.

The flat tension bars with a height of 10 mm and a thickness of 4 mm were designed in regard to the standard DIN 50125. At least 5 samples were tested with a test speed of 1.2 mm/min for statistic accuracy. The tests were carried out with a MTS servo hydraulic test machine ±100 kN of the company MTS Systems GmbH, Berlin, Germany.

Metallographic cuts were prepared with the Saphier 520 of the company ATM GmbH, Mammelzen, Germany, in order to investigate the structural changes due to the modified sintering strategy.

Scanning electron images (SEM) were taken with a Merlin VP COMPACT of the company Zeiss, Germany, in order to investigate the sinter quality of the test samples.

The porosity and density of at least three sintered samples were measured in accordance with the standard DIN EN 623-2.

The mechanical properties of SEBM manufactured parts can differ significantly in dependence of the oxygen content of the powder. A study has shown that used powder has higher oxygen content than new powder, which results in a decrease in the elongation of parts [13,14]. Therefore, an oxygen analysis was carried out with a TC-436 of the company Leco, St. Joseph, MI, USA. The carrier gas was helium.

**Figure 2:**
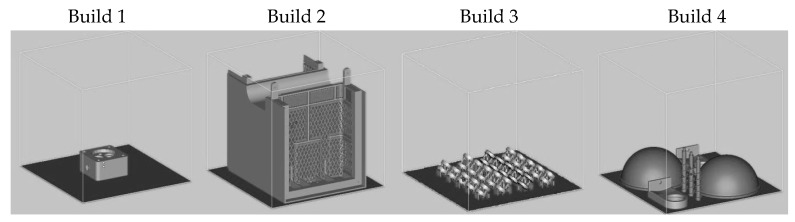
Different build jobs for build time investigation.

A variety of build jobs were carried out. Figure 2 shows the four build jobs that were used for the investigation of the build time. The criteria for these build jobs were build size, build volume, and the amount of parts needed in order to cover a large variety of different build jobs. For the four build jobs, Build 1 was categorized as one medium-sized component, Build 2 as one big component, Build 3 as many small components, and Build 4 as a mixture of small and large thin-walled components with a relatively low build volume. The build volume for the respective build jobs are as follows: Build 1 = 104.92 cm^3^, Build 2 = 693.12 cm^3^, Build 3 = 9.764 cm^3^, and Build 4 = 86.65 cm^3^.

## 3. Results and Discussion

**Figure 3:**
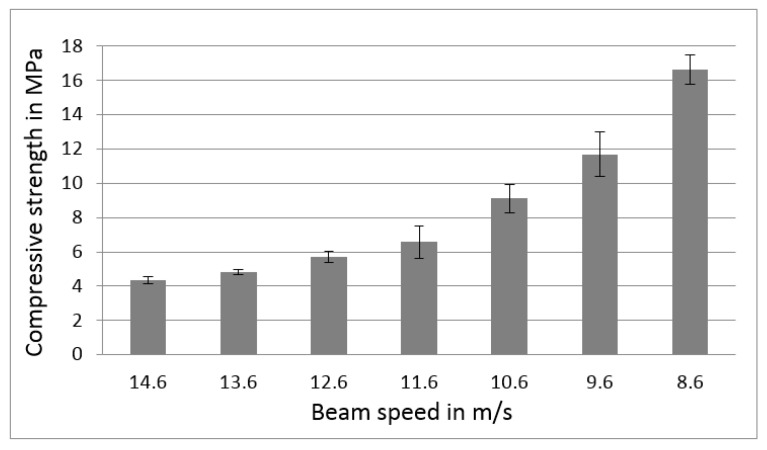
Results of the compressive strength test at different scan speed strategies.

For the experimental investigation, the EBM system A1 was prepared by positioning a stainless steel start plate, pulling a vacuum of at least 5 × 10^−4^ mbar for the chamber room, and adjusting the electron beam for highest accuracy. The corresponding themes for the processes (preheat theme and melt theme) were loaded with a layer thickness of 50 µm. The preheat theme was modified for the study, which consists of Preheat1 and Preheat2. The scan speeds used to manufacture the test cylinders were in a range from 14.6 m/s down to 8.6 m/s with an interval of 1 m/s. At 8.6 m/s, scattered repulsion effects were detected leading to the conclusion that, with lower scan speeds, the sintering process becomes more unstable and the powder starts to become blasted away due to the electrostatic effects. The manufactured compressive samples were tested, and the following results can be seen in Figure 3. It can be observed that the compressive strength follows an exponential increase with lower scan speeds. At a higher compressive strength, the powder has a higher connectivity, which results in better processability due to a lower chance of repulsion effects due to electrostatic charging. However, by lowering the scan speed too much and therefore increasing the energy input with electrons, the repulsion effects can overcome the connectivity of the powder. Hence, it is important to note that there is a process window in which a stable sintering works. By using different scan speeds, a certain process window for the Preheat2 theme was determined. There are other parameters such as energy input, line offset, line order, and focus offset that also have an impact on the process window and will be part of further investigations.

**Figure 4:**
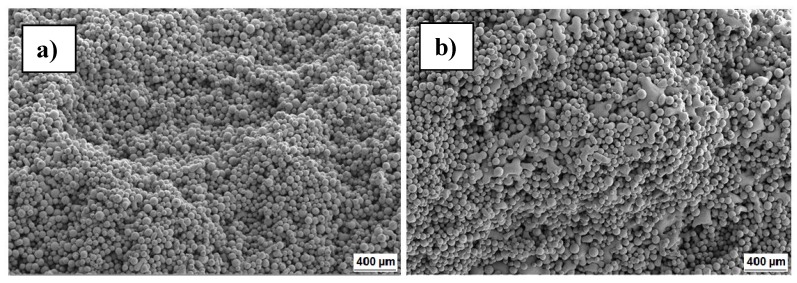
SEM images of samples magnified 30× with a scan speed of 14.6 m/s (**a**) and of 8.6 m/s (**b**).

SEM images underline the increased interconnectivity of the particles as shown in Figure 4. It is clearly visible that the powder morphology is starting to disappear at a scan speed of 8.6 m/s, indicating the beginning of a melting process. 

**Figure 5:**
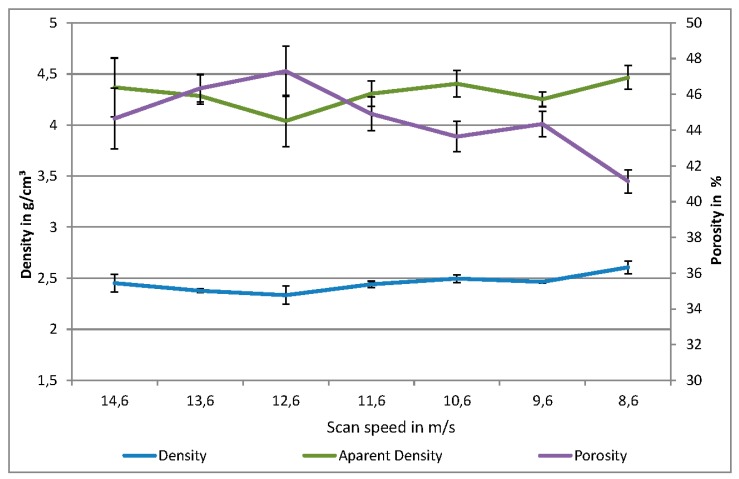
Results of the porosity measurement of different scan speed strategies.

Furthermore, porosity investigation has shown that the porosity decreases with decreasing scan speed, as depicted in Figure 5. It seems to follow a nearly linear trend, while the compressive strength increases exponentially. The deviations on the porosity can be explained by the superimposing effects of the scan speed and the cooling rate during sintering, leading to local densifications of the sample. This effect is dependent on the size and geometry of the part, since a large part exhibits lower cooling rates than a small part, resulting in a higher possible heat jam. The density measurement also shows a linear increase with decreasing scan speed, while the apparent density of the material roughly stays at the same value of 4.3 g/cm^3^.

**Figure 6:**
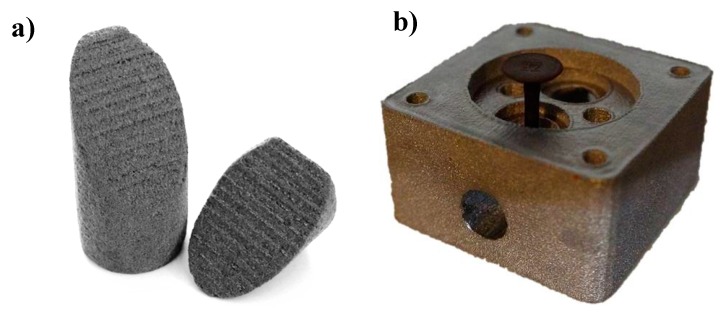
Sintered and tested compression sample with a scan rate of 8.6 m/s (**a**) and the component from Build 1 (**b**).

Figure 6 shows parts built only with the preheat theme, resulting in a highly porous structure. The parts were removed from the residual powder cake and were easy to handle for further investigation. 

With the idea of a stronger fused powder bed in localized areas, the standard Preheat2 strategy can be replaced by a modified strategy that only sinters the powder, which can then be fully melted with the subsequent corresponding melt theme. In doing so, the surrounding powder is easier to remove from the fully melted parts after the build job is done. Additionally, the modified strategy uses a decreased speed for the Preheat2 before the area is being fully melted. This was carried out in order to speed up build time. The original Preheat2 strategy scans a large area three times before starting to melt the defined area for the component. By using a slower scan speed for Preheat2, the repetitive scanning can be bypassed for a faster process. A scan speed of 9.6 m/s was chosen because the compressive strength showed a value that is about three times higher than the initial compressive strength, as seen in Figure 3. Therefore, the modified scan should result in a comparable compressive scan than the original scan, which runs three times in the original process.

The modified Preheat2 strategy showed a stable process with no powder blasting, which is a common problem [15,16]. With the modified Preheat2 strategy, samples for tensile tests were built and compared with specimens built with the original strategy.

As seen in Table 1, the tensile strength of the modified process is comparable to the original process. The tensile strength reaches 1001 (±16) MPa for the reference specimens, while the modified process results in specimens that reach a tensile strength of 1027 (±9) MPa.

These values correspond to the results of other studies [13,14,17]. However, the elongation (1.7% and 4.1%) is only a fraction of the value (~14%) determined by the above-mentioned studies. Furthermore, Koike et al. measured an SEBM product elongation of only about 2% [18]. They found out that a slight increase in tensile strength combined with a lower elastic modulus and higher brittleness could be explained by a higher amount of oxygen in the material, since the powder had been used several times prior to this study. Therefore, an oxygen analysis was carried out in order to quantify the oxygen content of the used powder. Titanium’s high affinity for oxygen is well-known. Additionally, the reaction between oxygen and titanium is increasingly rapid with increasing temperature. Over time, there is potential for the feedstock powder to change chemistry, specifically oxygen content [19]. Oxygen analysis showed that there was a high oxygen pickup. The nominal oxygen content of the new Ti6Al4V powder is 0.15%. In our measurements, while the powder used for the specimens had an oxygen content of 2880 ± 16 ppm (0.29%), which is twice as high as the nominal value. This can explain why the tensile bars exhibited such brittle behavior.

**Figure 7:**
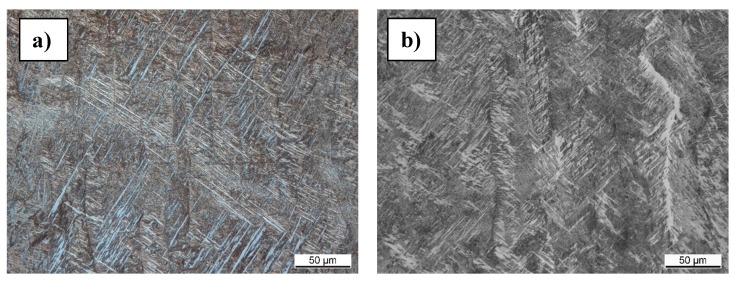
Metallographic cuts of a reference specimen (**a**) and a specimen with modified parameters (**b**).

Metallographic cuts were prepared in order to investigate and compare the structure of the specimens. Figure 7 shows the microstructure of a specimen with standard process parameters and modified parameters. Both microstructures show a lamellar and a very fine β grain phase, which leads to the conclusion that the modified parameters have little effect on the solidification process. 

The time reduction of the modified build strategy can vary in regard to different part geometries and dimensions as well as the number of parts. Table 2 depicts four exemplary build jobs with different components. In order to get a rough estimation of the time reduction, a large variety of 4 different build jobs have been used for simulating the build time. The simulation appoach was used. The results of the simulation show that the average build time lead to a time reduction of around 20% with values reaching up to 37%, as seen in Table 2. A general tendency of higher time reduction could not be observed in regard to part size, geometry, or amount of parts.

The simulated build time differs from the real build time due to factors such as variations in adding of a powder layer, occasional arc trips, or the preheating time of the start plate.

## 4. Conclusions

The modification of the sintering process during electron beam melting led to a significant improvement in productivity. A decrease in scan speed during the sintering process revealed an exponential increase in compressive strength of the sinter cake specimens, which can be used to accelerate the sintering and melting process. Additionally, the sintered powder can be removed from final melted parts more easily. The local sintering prior to the melting process of the desired part has shown an increase in build efficiency between 14% and 37%, depending on the build geometry, size, and number of parts. However, it was not possible to find a correlation between these aspects since the original sintering process follows a complex algorithm in order to calculate the sintering area, which also depends on the aforementioned aspects as well as the location of the parts. Mechanical properties remained comparable. Oxygen analysis showed that the Ti6Al4V powder had high oxygen content, resulting in a high brittleness compared with fresh powder. The metallographic cuts of the samples show no noticeable change in structure. The results show that the modification of the sintering process can dramatically improve the SEBM technology in regard to time efficiency. By adjusting the sinter parameters, such as energy input and focus offset, productivity can be increased even further. The SEBM technology still has great potential for improvement in all areas by adjusting the parameters that are involved in all process steps.

## Figures and Tables

**Table 1:** Mechanical properties.

Properties	Tensile Strength (MPa)	E-Modulus (GPa)	Elongation (%)
Reference	1001.1 ± 16.0	106.3 ± 4.0	1.7 ± 0.2
Modified	1026.6 ± 9.1	107.4 ± 9.0	4.1 ± 0.1

**Table 2:** Time duration of exemplary build jobs.

Build	Standard Parameters (min)	Modified Parameters (min)	Time Reduction (%)
Build 1	344	217	37
Build 2	1780	1300	27
Build 3	234	170	27
Build 4	437	377	14
